# A case study of the utilization of clozapine treatment for treatment‐resistant schizophrenia associated with 22q11.2 deletion syndrome

**DOI:** 10.1002/npr2.12333

**Published:** 2023-03-16

**Authors:** Arisa Tsurue, Hideki Funahashi, Keiichi Tsurue, Masahiko Kawano, Yasushi Ishida, Yoji Hirano

**Affiliations:** ^1^ Miyakonojo Shinsei Hospital Miyakonojo Japan; ^2^ Department of Psychiatry, Faculty of Medicine University of Miyazaki Miyazaki Japan

**Keywords:** 22q11.2 deletion syndrome, Asian, clozapine, treatment‐resistant schizophrenia

## Abstract

**Background:**

The optimal treatment strategy for patients with treatment‐resistant schizophrenia (TRS) associated with 22q11.2 deletion syndrome (DS) remains a subject of debate.

**Case Presentation:**

We present the case of a 40‐year‐old female patient diagnosed with TRS and 22q11.2DS who was effectively treated with clozapine. She was diagnosed with schizophrenia and mild intellectual disability during her adolescence; despite being hospitalized for a period of 10 years beginning in her 30s, she continued to exhibit symptoms of impulsivity, and explosive behavior, requiring periods of isolation. We ultimately decided to switch her medication to clozapine, which was administered with caution and gradually titrated upward, with no discernable adverse effects, resulting in a marked improvement in her symptoms and obviated the need for isolation. Subsequently, the patient's history of congenital heart disease and facial abnormalities prompted initial suspicions of a 22q11.2DS diagnosis, which was subsequently confirmed through genetic testing.

**Conclusion:**

Clozapine may serve as an efficacious pharmacological intervention for TRS patients with 22q11.2DS, including those of Asian descent.

## Abstract 2

Clozapine may serve as an efficacious pharmacological intervention for treatment‐resistant schizophrenia patients with 22q11.2 deletion syndrome, including those of Asian descent.

## Graphical abstract

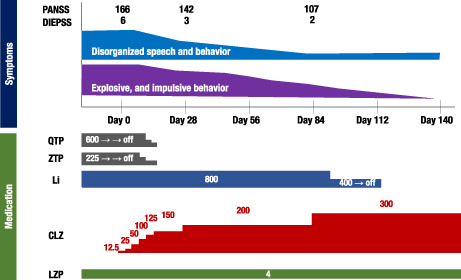

## 1 INTRODUCTION

Chromosome 22q11.2 deletion syndrome (DS) is the most prevalent microdeletion disorder in human populations, with a prevalence of approximately 1 in every 4000–5000 live births.^1^, ^2^ A definitive diagnosis is typically established by detecting the deletion of the 22q11.2 chromosomal region through the use of fluorescence in situ hybridization (FISH). Individuals with this genetic condition have diverse phenotypes, with over 180 clinical manifestations that affect every organ system.^1^ The major clinical manifestations of 22q11.2DS include congenital heart defects (CHDs), palatal anomalies, immune deficiency, hypoparathyroidism/hypocalcemia, subtle dysmorphic facial features, short stature, and minor deformation of the auricle.^1^

Individuals with 22q11.2DS exhibit a markedly elevated risk of developing psychiatric disorders including schizophrenia and intellectual disability.^2^ Since the 1990s, with the advent and popularization of the FISH method, the number of cases diagnosed during childhood has increased.^2^ Considering the prevalence of 22q11.2DS, it appears that there is a substantial number of patients with psychotic disorders who remain undiagnosed.

The neurobiological mechanisms and optimal treatment strategy for schizophrenia, particularly treatment‐resistant schizophrenia (TRS), remain equivocal.^3^, ^4^, ^5^, ^6^, ^7^, ^8^, ^9^, ^10^, ^11^, ^12^ Moreover, the most efficacious treatment approach for TRS associated with 22q11.2DS is a subject of debate.^13^, ^14^ Notably, conventional antipsychotics and mood stabilizers are known to have little effect on the psychotic symptoms of 22q11.2DS,^15^, ^16^ and thus, the expeditious development of a pharmacological intervention that is efficacious for TRS patients with 22q11.2DS is imperative. However, there are a limited number of studies from Western countries that have examined the efficacy of clozapine in this population.^13^, ^14^ Furthermore, to our knowledge, there are no reports from Asia. In light of the diverse biological variations, it is important to document cases of 22q11.2DS not only in individuals of European descent but also in Asian populations, including Japanese individuals.^17^

Here, we report our experience with the successful utilization of clozapine treatment for TRS associated with 22q11.2DS.

## 2 CASE PRESENTATION

**FIGURE 1:**
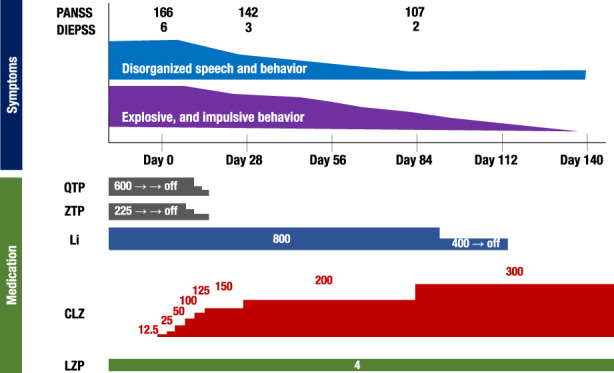
Clinical course of the patient diagnosed with TRS associated with 22q11.2 DS. Abbreviations: CLZ, clozapine; Li, lithium; LZP, lorazepam QTP, quetiapine; ZTP, zotepine (medication dose: mg/day)

A 30‐year‐old Japanese female was hospitalized due to severe psychotic symptoms. The patient, who was delivered normally at full term, was diagnosed with CHD after birth and subsequently underwent surgical intervention. The postoperative course was uneventful. Despite being identified as mildly intellectually retarded in kindergarten, she was able to attend regular classes with the aid of her teachers and peers. Throughout her childhood, she displayed no discernible symptoms or traits indicative of attention deficit hyperactivity disorder (ADHD) or autism spectrum disorder (ASD). Following her high school graduation, she contributed to her family's business. As a teenager, the patient began experiencing exacerbated hallucinatory and delusional symptoms without provocation. Despite multiple admissions to a nearby psychiatric hospital and treatment with various antipsychotic medications, her symptoms persisted. At the age of 30 years, despite ongoing pharmacotherapy (with a maximum dose of haloperidol, chlorpromazine, and risperidone), the patient had persistent hallucinations, delusions, incoherent speech, and explosive and impulsive behavior, as well as aggressive behaviors such as yelling at, biting, and kicking her mother. As a result, she was admitted to our hospital for further treatment. Her electrocardiogram revealed sinus tachycardia (102/min). Owing to the patient's marked level of restlessness, an electroencephalogram could not be administered. No other abnormalities including parathyroid hormone and calcium level were present in laboratory findings. Upon admission, the patient was diagnosed with schizophrenia and mild intellectual disability (IQ = 68). Despite high doses of various second‐generation antipsychotics such as olanzapine (20 mg) and risperidone (8 mg), the patient continued to exhibit agitation, disorganized speech, and behavior. The patient's level of functioning, including interpersonal relations and self‐care, significantly diminished, and she required constant assistance with activities of daily living such as toileting, bathing, and eating. Additionally, the patient exhibited behaviors such as undressing in public, excessive drinking, and sudden, violent outbursts, which often necessitated isolation. As a result, she was unable to leave the hospital for a period of 8 years. When she was 38 years old, we ultimately decided to switch her medication to clozapine. Prior to the initiation of clozapine treatment, the patient had been taking quetiapine 600 mg/day, zotepine 225 mg/day, lithium 800 mg/day, and lorazepam 4 mg/day. Quetiapine and zotepine were gradually tapered off, and clozapine was started at a dose of 12.5 mg/day, which was titrated up to 200 mg/day on day 28. Subsequently, the patient's violent behavior, disorganized speech, and behavior gradually decreased. On day 84, the clozapine dose was increased to 300 mg/day, while the lorazepam dose of 4 mg/day was continued and lithium and flunitrazepam were discontinued. As a result, the patient's severe violent behavior disappeared, and she spent more days peacefully, without the need for behavioral restrictions. The Positive and Negative Syndrome Scale (PANSS) score decreased from 166 to 107, and the Drug‐Induced Extrapyramidal Symptoms Scale (DIEPSS) score decreased from 6 to 2 (Figure.1). Periodic assessments for potential side effects were performed. When the patient was 40 years old, a specialized team from our hospital and a university hospital conducted a review of her case and reached the conclusion that the patient could have 22q11.2DS, based on the following physical features: psychiatric symptoms that diverged from those of typical schizophrenia, such as impulsive and explosive behaviors, which were more prominent than hallucinations and delusions; a history of tetralogy of Fallot; subtle dysmorphic facial features and an open nasal cavity. After informing the patient and her family about the proposed chromosome examination and obtaining written consent, a deletion analysis of chromosome 22 was performed, which revealed a deletion in the 22q11.2 region, thereby confirming the diagnosis of the disease in conjunction with the patient's clinical symptoms.

## 3 DISCUSSION

### 3.1 22q11.2 DS and psychiatric disorders

Individuals with 22q11.2DS have a higher risk of psychiatric complications than the general population, particularly schizophrenia, neurodevelopmental disorders, anxiety disorders, and mood disorders.^1^ ADHD and anxiety disorders are present in 30–40% of children with this condition; 20–30% of adolescents with 22q11.2DS may develop autism spectrum disorder, and 30–40% of adults may develop schizophrenia later in life.^18^ The reported mean full‐scale IQ score was 71.25 (SD = 12.13). The mean age of onset of schizophrenia was 17.7 years and the prevalence of schizophrenia is high among all age groups and increases with age, with a particularly high rate of 41% in the group aged 36 years and older.^2^, ^19^ Copy number variation (CNV) is known to be an important genetic factor in the development of schizophrenia, and among CNVs, the 22q11.2 deletion is considered a representative risk factor for the development of psychosis.^1^ Patients with 22q11.2DS show higher rates of explosive and impulsive behavior than those with idiopathic schizophrenia.^20^ Our patient exhibited highly explosive and impulsive behavior and presented significant challenges in management over an extended period of time. The psychiatric symptoms associated with 22q11.2DS are varied and require ongoing psychiatric screening and follow‐up throughout an individual's life, which may sometimes lead to treatment resistance, as was the case with this patient.

### 3.2 Treatment with clozapine

#### 3.2.1 General precautions

Clozapine is widely regarded as the gold standard for TRS treatment and has been shown to improve psychotic symptoms, negative symptoms, rehospitalization rates, and long‐term mortality.^21^ However, clozapine is associated with a low but significant risk of serious side effects, such as agranulocytosis and myocarditis. In Japan, clozapine is strictly controlled and can be administered only by medical institutions registered with the monitoring service and only when all necessary blood tests have been administered and other criteria have been met. Based on a national database of over 9000 Japanese patients with TRS, Imazu et al. analyzed physical adverse events leading to medication discontinuation. Neutropenia and leukopenia showed the highest incidence rate, with an interquartile range (IQR) of 14.3 (7.3–45.6) weeks for onset, requiring early attention. Cardiac problems and seizures were observed relatively early in treatment with IQRs of 3.9 (2.9–9.4) and 17.9 (5.9–38.8) weeks, respectively, while gastrointestinal disorders were observed with an IQR of 38.9 (8.9–116.4) weeks, suggesting that long‐term monitoring is needed.^22^ Matsui et al. reported the need to monitor patients for the emergence of agranulocytosis for approximately 1 year after initiating clozapine.^23^

#### 3.2.2 Special considerations for schizophrenia patients with 22q11.2 DS

There are several reports from Western countries on the use of clozapine in TRS patients with 22q11.2DS, with seizures being a particularly concerning side effect.^13^, ^14^, ^24^ Hypocalcemia has been identified as a potential trigger for seizures in adults with 22q11.2DS and the use of antipsychotics and antidepressants should be considered potential contributing factors. Similar triggers may be responsible for seizures in patients treated with clozapine.^25^ Therefore, in schizophrenia patients with 22q11.2DS, the initial dosage should be administered with caution. In the present case, the dosage of clozapine was incrementally increased with moderation, as opposed to starting with the recommended dosage. Furthermore, other medications, such as lithium, which have the potential to lower the seizure threshold, were discontinued as much as possible. The patient's condition subsequently improved without adverse effects. Valproic acid is often used for clozapine‐induced seizures;^26^ however, its concomitant use should be avoided because it impairs the clearance of clozapine. Furthermore, the utilization of valproic acid during the early stages of clozapine administration increases the incidence of myocarditis and granulocytopenia.^27^

### 3.3 Significance of diagnosis and follow‐up

The symptoms and severity of 22q11.2DS are diverse and variable among affected individuals, despite the deletion being largely similar;^1^ therefore, it may be diagnosed at birth or go undetected until adulthood. Indeed, the symptoms leading to diagnosis are age dependent: from birth to 2 years of age, cardiac disease, immunodeficiency, and hypocalcemia are commonly observed; from 2 years of age through school age, learning disabilities and dysarthria due to nasopharyngeal obstruction dysfunction and submucosal cleft palate are commonly observed; and from adolescence through adulthood, learning disabilities, latent hypocalcemia, and psychiatric disorders are prevalent.^1^ Upon comparing this case with the scant number of case reports from Japan,^28^ it is evident that agitation and intellectual disability are prevalent, albeit with varying ages of diagnosis and symptomatic presentations among cases. Given the prevalence of 22q11.2DS and the incidence of psychiatric disorders, it is likely that many patients with undiagnosed 22q11.2DS present to psychiatrists during adolescence and adulthood and receive treatment without being aware of their diagnosis. In the present case, a definitive diagnosis was made 10 years after the patient's first visit to our hospital and more than 20 years after her first psychiatric consultation. Reports of psychosis associated with 22q11.2DS from a single private psychiatric hospital such as ours are extremely rare in Japan.^28^ The FISH method, which is required for definitive diagnosis, can be outsourced for a cost of 30 280 yen in Japan. Confirming a 22q11.2DS diagnosis has several advantages, including the ability to monitor for potentially associated findings and diseases such as lifelong hypocalcemia, otolaryngological disorders, and juvenile Parkinson's disease. The accumulation of patient data is of significance, as psychiatric issues will continue to be involved at a high rate in the future. In the last 30 years, the understanding of 22q11.2DS has improved in urban areas in Japan. However, currently, only a limited number of patients are able to benefit from this treatment. Given that 22q11.2DS and mental, physical, and intellectual disabilities coexist to varying degrees,^1^, ^2^ the difficulties faced by patients and their families are diverse and varied; thus, it is impossible for psychiatrists alone to provide support for these individuals. We hope to reduce disparities in medical care across regions by reinforcing cooperation among pediatric, psychiatry, internal medicine, and other physical departments, as well as among healthcare, welfare, and educational organizations. Additionally, by deepening our knowledge of 22q11.2DS, as described in this report, we aim to improve the quality of care provided to patients with this condition.

## AUTHOR CONTRIBUTIONS

AT, KT, and MK treated the patient. AT wrote the first draft. HF, YI, and YH critically revised the manuscript. All authors have read and approved the final manuscript.

## FUNDING INFORMATION

This research was supported in part by JSPS KAKENHI Grant Number, JP20KK0193 (YH) and JP21H02851 (YH).

## CONFLICT OF INTEREST STATEMENT

The authors declare that they have no conflicts of interest.

## ETHICS STATEMENT

Approval of the research protocol by an institutional review board: N/A.

Informed consent: The patient and her family provided informed consent for the publication of this case report.

Registry and the registration no. of the study/trial: N/A.

Animal Studies: N/A.

## Data Availability

Data sharing not applicable to this article as no datasets were generated or analysed during the current study.
